# Aerial and aquatic biological and bioinspired flow control strategies

**DOI:** 10.1038/s44172-023-00077-0

**Published:** 2023-05-26

**Authors:** Ahmed K. Othman, Diaa A. Zekry, Valeria Saro-Cortes, Kyung Jun “Paul” Lee, Aimy A. Wissa

**Affiliations:** Department of Mechanical and Aerospace Engineering, Princeton University, Princeton, NJ 08544 USA

**Keywords:** Mechanical engineering, Physiology

## Abstract

Flow control is the attempt to favorably modify a flow field’s characteristics compared to how the flow would have developed naturally along the surface. Natural flyers and swimmers exploit flow control to maintain maneuverability and efficiency under different flight and environmental conditions. Here, we review flow control strategies in birds, insects, and aquatic animals, as well as the engineered systems inspired by them. We focus mainly on passive and local flow control devices which have utility for application in small uncrewed aerial and aquatic vehicles (sUAVs) with benefits such as simplicity and reduced power consumption. We also identify research gaps related to the physics of the biological flow control and opportunities for device development and implementation on engineered vehicles.

## Abstract (web summary)

Ahmed Othman and colleagues review flow control strategies used by birds, insects and aquatic animals, and how they have been adopted in engineered devices. The focus on passive and local flow control is of particular relevance to the design of small uncrewed aerial and aquatic vehicles.

## Introduction

**Fig. 1:**
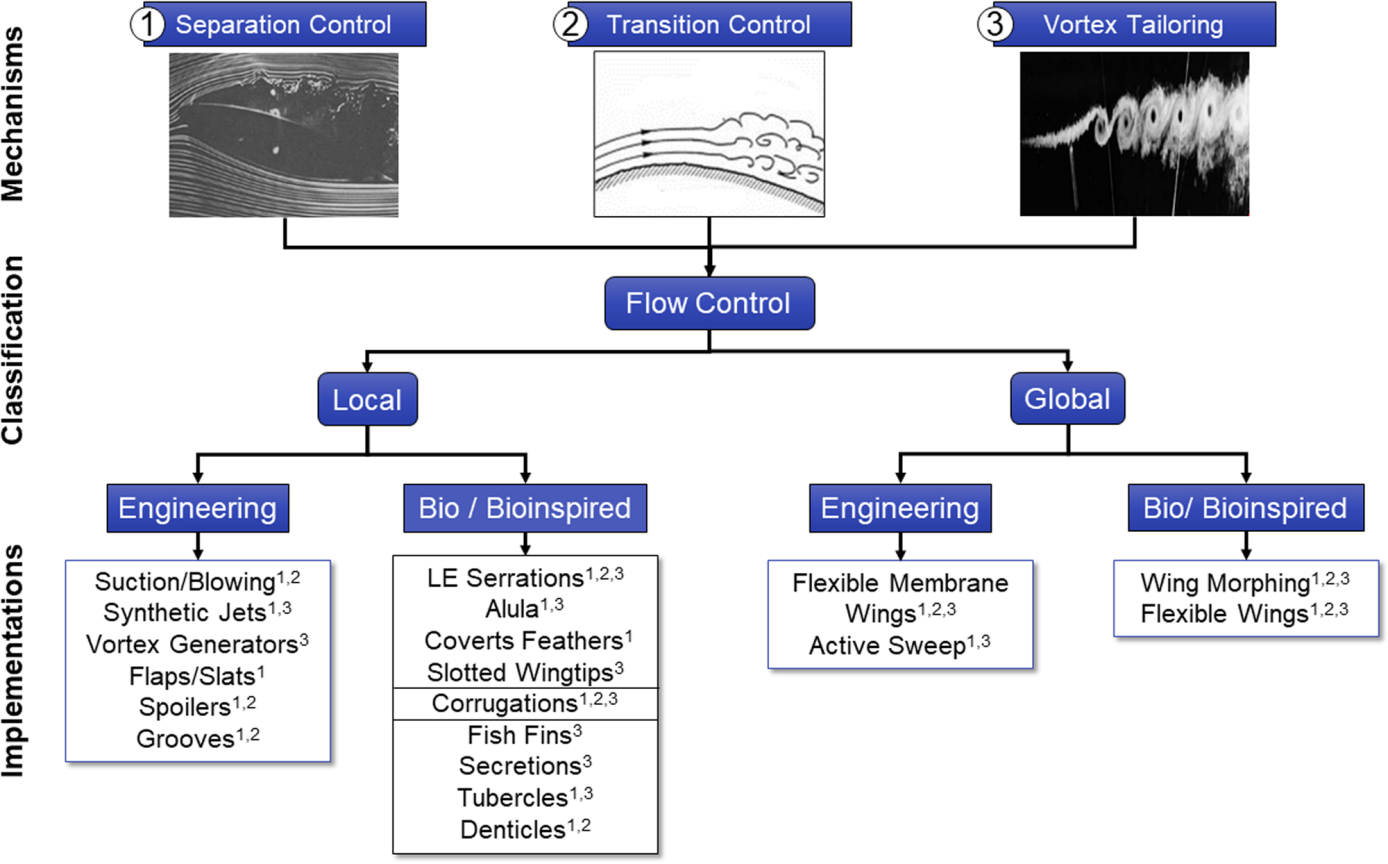
Flow control mechanisms and classifications and their implementations as flow control devices. The superscripts indicate the flow control mechanism implemented by each flow control device. The figure classifies flow control mechanisms into 1- separation control [Reproduced from ref. ^160^, The National Aeronautics and Space Administration (NASA)]^160^, 2- transition control [Reproduced from ref. ^161^, The National Aeronautics and Space Administration (NASA)]^161^, 3- vortex tailoring [Reproduced from ref. ^162^, The National Aeronautics and Space Administration (NASA)]^162^.

Natural flyers and swimmers operate under various conditions. The same animal, for example, a flyer, can repeatedly take off, hover, glide, flap, and perch in different environmental conditions, such as during gusts and thermals or closer to the ground or the water surface. This agility and maneuverability are possible because of the ability of these biological systems to alter the flow around their lifting and thrusting surfaces, using what is referred to as flow control. Flow control is defined as attempting to favorably modify a flow field’s characteristics compared to how the flow would have developed naturally along the surface^1,2^. Flow control mechanisims include postponing boundary layer separation, delaying or advancing laminar-turbulent transition, and altering vortical structures and dynamics (Fig. 1). Such mechanisms often lead to drag reduction, lift enhancements, or noise suppression and can be implemented through flow control devices such as flaps, slats, or synthetic jets, to mention a few.

Multiple classifications exist for flow control devices; one of the most common ways is to classify flow control techniques as active or passive based on their energy expenditure. Passive flow control requires no actuation nor energy expenditure, making it less complex and more affordable; however, it cannot always provide enough control authority or adaptability, especially in complex applications^3,4^. On the other hand, active flow control devices require actuation and power to affect the flow^5^. Another classification is related to the area affected by the flow control device, namely local versus global flow control (Fig. 1). Local flow control devices introduce localized changes to the flow structures at a particular area of the lifting surface. In contrast, global flow control devices usually affect the flow over the entire wing or lifting surface. Examples of local flow control devices include synthetic jet actuators or roughness strips, while wing sweep and camber morphing systems would be considered global flow control devices.

There have been extensive studies and reviews on flow control techniques and devices^3,4,6,7^. Most of these studies focus on engineered flow control devices, large-scale aircraft, and fully developed turbulent conditions (Reynolds number (Re) ≥ 10^6^)^8^. More recently, studies have focused on bioinspired and biological flow control techniques^9^. Understanding biological and bioinspired flow control techniques help in two crucial ways. First, these techniques provide new insight into biological locomotion and uncover the physics that enable these organisms to be adaptable and efficient across multiple flight conditions. Second, bioinspired flow control techniques are more suitable for small-scale uncrewed vehicles than traditional approaches developed for large-scale and high-speed operations. Due to the limited volume and payload capabilities of small-scale vehicles, traditional flow control devices’ size, complexity, and weight penalties are prohibitive^1,6,7,10,11^. Scaling down conventional flow control devices may also not be suitable for the mission demands of small-scale vehicles. Traditional flow control techniques are most effective over a limited range of angles of attack and operational conditions. In contrast, the mission demands and applications of small uncrewed aerial or aquatic vehicles (sUAVs) often require them to operate over a wide range of angles of attack. Thus, biological flow control techniques offer a suitable solution to augment the performance of sUAVs because of the similarities between the operational conditions of these vehicles and the biological organisms.

This article reviews biological flow control techniques and devices implemented by birds, insects, and aquatic animals and the engineered systems inspired by them. The article expands on recent review articles focused on biological flow control^9,12^ by including the biological flow control mechanisms and the bioinspired devices inspired by them, highlighting key research gaps and opportunities for sUAVs. The article is divided into three main sections: flow control in avian and avian-scale fliers, flow control in insect and insect-scale fliers, and flow control in marine animals and bioinspired aquatic systems. For each section, the article categorizes the flow control devices according to well-defined flow control mechanisms and classifications (Table 1). The article mainly focuses on passive and local flow control devices because they can be easily integrated into small engineered vehicles compared to active and global flow control devices. Finally, the article highlights some of the gaps in the literature and the exciting opportunities for bioinspired flow control research.

**Table 1:** Summary of selected biological and bioinspired flow control devices studies.

The abbreviations under the study type are as follows: E is an experimental study, F is a free-flight experiment, and S refers to numerical simulations. The icons in the flow control mechanism column are the same icons used in Fig. 1.

## Avian flow control

Bird wings have multiple sets of feathers that serve numerous functions, from thermal insulation to improved flight performance and sound damping^13–15^. Aerodynamically, aside from generating the necessary aerodynamic forces for flight, feathers also serve as flow control devices^15–18^. A detailed description of bird wing geometry, internal and external structures, and feathers’ form and function can be found in^15^. This section reviews four feather systems or feather features for flow control, namely the covert feathers, the alula, the emargination of the primary feathers that create slots towards the wingtips, and leading edge serrations that have been mainly observed in owl feathers.

### Covert feathers

**Fig. 2:**
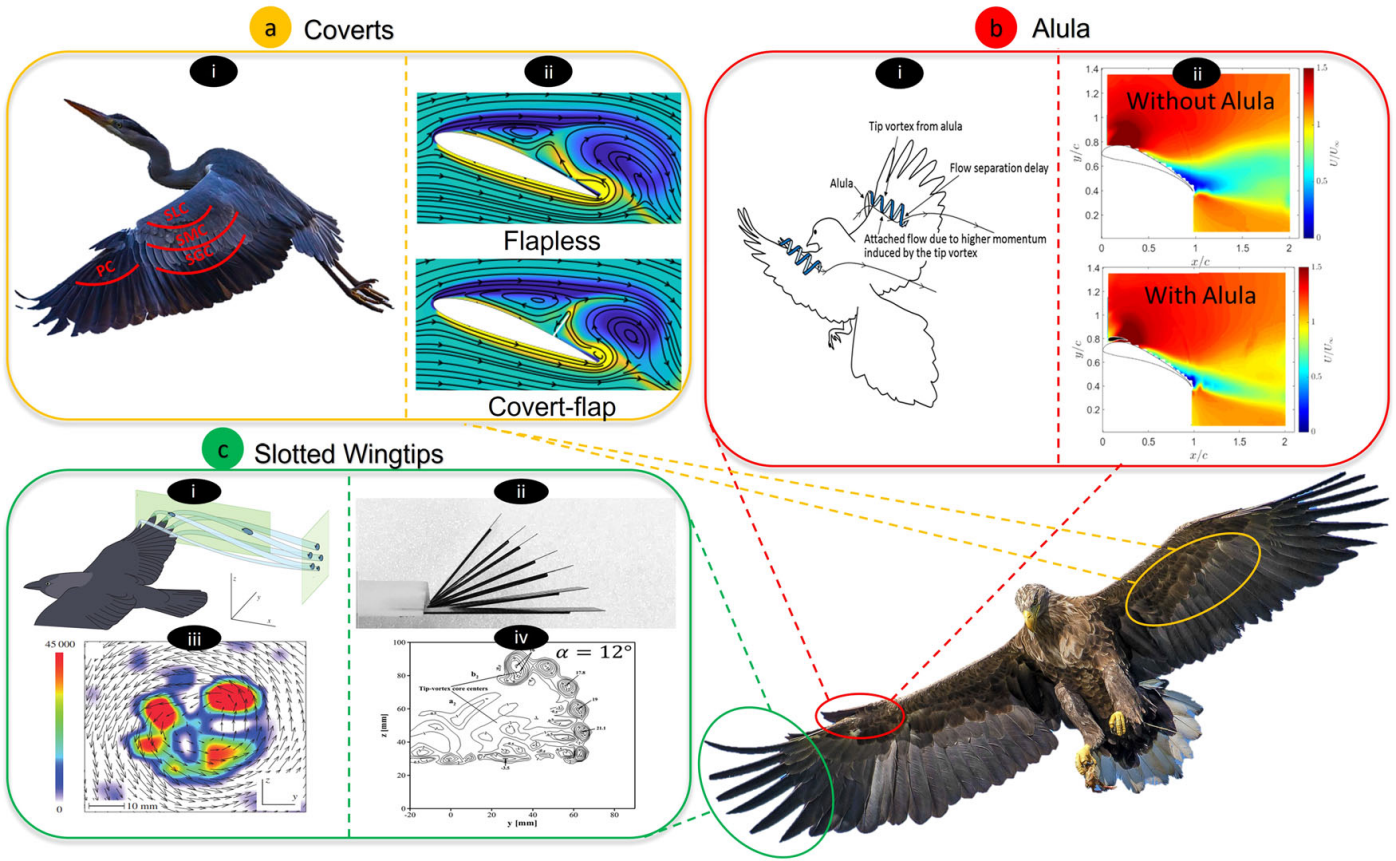
Selected avian and avian-scale flow control devices inspired by feathers. Selected feather systems shown here are the coverts (**a**), the alula (**b**), and the slotted wingtips (**c**). **a**–i Coverts on the upper surface of a heron wing where the initials PC, SLC, SMC, and SGC stand for Primary Coverts, Secondary Lesser Coverts, Secondary Median Coverts, and Secondary Greater Coverts, respectively. [Source: Pixabay] **a**-ii Numerical vortex contours show the streamlines superimposed with the vorticity contours. Blue and yellow contours represent clockwise and counterclockwise vorticity, respectively. The figure shows that the covert-inspired flaps can block reverse flow and enhance lift^29^. [Adapted with permission from ref. ^29^] **b**-i The alula in birds generates tip vortices, which delay separation and boundary layer reversal^42^. [Reproduced from ref. ^42^]. **b**-ii An alula-inspired device applied to an engineered wing is also effective at delaying separation compared to a wing without an alula, as indicated by Particle Image Velocimetry (PIV) results^43,45^. [adapted with permission from ref. ^45^] **c** Flow visualization of vortex structures around the slotted wing tip of a jackdaw (i and iii) shows the effect of slots on the vortex structures at the wingtips [adapted with permission from ref. ^54^] with similar studies performed on engineered wings showing similar vortex structures (ii and iv) [adapted with permission from ref. ^65^].

The covert feathers are a set of contour feathers that exist on the upper and lower surfaces of birds’ wings and tails. Wing coverts consist of multiple overlapping rows or layers, where the row near the leading edge is named the lesser coverts, followed by the median coverts and the greater coverts (Fig. 2a-i)^19^. Understanding the role of the coverts in avian flight as a flow control device was crucial for the development of similar bio-inspired flow control devices^16^. In a study done by Carruthers et al.^17^, they examined the coverts deployment of a steppe eagle (*Aquila nipalensis*) during different flapping and perching sequences both indoors and outdoors using a high-speed digital video camera placed on the upper wing. In their study, the coverts’ response was classified as a passive aeroelastic response, since the bird muscles can only apply force at the base of those feathers, while during the experiments, the deployment of the coverts was initiated from the feather tip^17^. The upperwing and underwing coverts were noticed to deploy during high-angle of attack maneuvers, such as perching, take-off, landing, and gust maneuvers. Carruthers et al. also observed that the underwing coverts deploy at take-off, landing, and flapping perching sequences. The underwing coverts, highlighted by the orange circle in Fig. 2, were noticed to deploy all together as one unit, which makes them analogous to Kruger flaps. Kruger flaps protrude from the leading edge of engineered wings to augment and extend the lift curve slope^20^. They are typically used during take-off and landing, which is also when the underwing coverts were noticed to deploy. On the other hand, the greater upperwing coverts (Fig. 2a-i) were noticed to deploy during the downstroke of flapping flight and in response to gust. Moreover, secondary upperwing coverts were noticed to deploy during both gliding and flapping perching sequences. The study suggests that the upperwing and underwing coverts appear to be used for flow control to enhance unsteady maneuvers, and may also provide sensory feedback to the bird^17^.

Due to their simplicity and aerodynamic advantages, the coverts were adapted on engineering wings in multiple forms to obtain a passive yet adaptive high-lift flow control device for stall mitigation (Fig. 2a-ii). Engineering studies have expanded the parameter space beyond what is in nature; for example, while in birds covert feathers are limited to the front half of the wing, engineering studies experimented with covert-inspired flaps at different locations ranging from the leading edge of the wing to the trailing edge. Moreover, the implementation of covert-inspired devices in engineering studies ranges from real feathers and hair-like flaps to rigid plastic and metal flaps with various geometries^21–24^. Furthermore, flaps with various mobility have also been studied, starting from simple static flaps at a fixed angle, to freely moving flaps, to torsionally-hinged flaps with adjustable stiffness^25–30^. Despite the different structural and mobility forms of the covert-inspired flaps, most studies confirmed that upperwing coverts act as a lift enhancement flow control device at post-stall conditions.

Most of the engineering studies have focused on upperwing covert-inspired devices. Duan and Wissa^25^ showed that rigid static covert-inspired flaps placed between 40% and 80% of the chord from the leading edge can improve post-stall lift up to 23% at a *Re* ~ (10^5^). Several studies ^26,27,29,31^ also examined the effects of freely moving upperwing flaps at *Re* ~ (10^5^−10^6^). Bechert et al.^27,28^, and Meyer et al.^31^ examined the effects of a freely moving flap on two different airfoils using both wind tunnel testing and numerical simulations and observed lift improvement of more than 10% and stall delay in both studies. They attributed the lift enhancement of the trailing edge coverts to the flap blocking the reverse flow and adverse pressure from advancing towards the leading edge of the airfoil in what they called a “pressure dam” effect. Coverts-enabled lift enhancement was also evident at a wide range of laminar low Reynolds number studies, *Re* ~ (10^3^−10^4^) both numerically and experimentally^30,32,33^. Nair and Goza^33^ and Othman et al.^29^ analyzed the fluid-structure interaction of an upperwing torsionally hinged covert-inspired flap at two different Re regimes (*Re* = 1000 and *Re* = 200, 000) to understand the effect of different flap structural properties (i.e., flap inertia and hinge stiffness) on the vortex structure and shedding frequency. Their study shows that low-inertia flaps were more effective at improving lift than high-inertia flaps. They also found that there are several similarities between both Reynolds numbers. For example, the covert-inspired flaps do not alter the vortex shedding frequency of the original airfoil at both high and low Re flows, with some unsteadiness for the high Re regimes due to turbulent transition^29^. Thus, despite the difference in the flow physics between laminar [$Re={{{{{{{\mathcal{O}}}}}}}}(1{0}^{3})$] to transitional [$Re={{{{{{{\mathcal{O}}}}}}}}(1{0}^{4}-1{0}^{5})$] to turbulent flows [$Re={{{{{{{\mathcal{O}}}}}}}}(1{0}^{6})$], the effectiveness of the covert-inspired flaps in improving lift across Reynolds numbers regimes suggests their suitability as a flow control device for different flight regimes and different UAV configurations^24,32–34^.

Fewer studies have considered the role of multiple upperwing covert-inspired flaps and underwing devices. Duan and Wissa studied the effect of multiple flaps arranged chordwise on a 2D airfoil at a *Re* ~ (10^5^), and they found that the flaps have an additive effect on lift post-stall where the lift improvements increased with the number of flaps^35^. Nair et al.^36^, also studied the effect of multiple flaps numerically at a much lower Re ~ (10^3^), and found that at this low Re deploying a single flap near the trailing edge alone results in the greatest lift improvement compared to the multiple flap cases. As for underwing covert-inspired devices studies, an experimental investigation by Wang et al. compared the aerodynamic effects of flaps, made from real feathers, on the upper and lower sides of a NACA 0012 airfoil at *Re* = 30, 000. The flap mounted on the suction (upper) side was found to have a positive impact at post-stall angles. On the other hand, when mounted on the pressure (lower) side, the flaps show aerodynamic benefits at small angles of attack (*α* = −4^∘^ to 8^∘^). Using PIV measurements, the authors attributed the lift benefits of the pressure side flaps to the increase in leading-edge vorticity and the larger dead flow region in the vicinity of the flapped airfoil pressure surface, leading to generation of high pressure and greater downward flow momentum beneath the pressure surface for the flapped case^24^.

In addition to wind tunnel experiments and numerical simulations, Meyer et al.^31^ performed free-flight experiments to test the effectiveness of the covert-inspired flaps during actual flight. Freely moving flaps were mounted on top of a STEMME S10 motor glider at *Re* = 10^6^. It was found that the flap improved maximum lift by about 10% at post-stall angles of attack, which matches the results of both wind tunnel experiments and simulations. Pilot comments indicated that changes to flight behavior were moderate when the flaps were mounted at the inner part of the wing and that keeping flight speed near stall values was easier with the movable flaps attached. On the other hand, the same study examined the use of covert-inspired flaps on swept and tapered wings, and it was found that in these cases, flow separation is dominated by 3D secondary flows, which makes these flaps less effective. Moreover, the flaps were found not to be compatible with vortex generators upstream of the flaps; this is because the vortex generators generate wrapped free shear layers with which the flaps cannot interact in a meaningful way. Furthermore, under attached flow conditions (small angles of attack), the freely moving flaps get slightly raised, which results in a slight drag increase and a lift detriment due to the small separation regime at the end of the flap.

In summary, covert feathers and covert-inspired systems can be considered high-lift devices that can be used for separation control during high angle of attack maneuvers to enhance post-stall lift. They improve lift by blocking the reverse separated flow from propagating upstream of the wing. We classified the coverts flow control mechanism as separation control across all Reynolds numbers regimes (Table 1) However, despite many studies on covert-inspired flow control devices, there are still gaps that warrant further investigation. For example, there is a need for more studies focused on the interaction between upperwing and underwing coverts as a function of the flow conditions and the flaps’ properties. Furthermore, the interaction of covert-inspired flaps with traditional flow and flight control techniques such as flaps, ailerons, and slats has not been studied. Finally, covert-inspired devices have yet to be implemented on small-scale UAVs during flight to evaluate their benefits and downsides critically and assess how these flaps change the wing’s stability parameters and the aircraft’s propulsive efficiency.

### Avian alula

Another feather system investigated for its role in flow control is the alula. The alula is a collection of feathers on the hand-wing of most birds^15,37^. Structurally, the alula is a skeletal bone digit named the alular digit. It is attached to the carpometacarpus bone on the wing^15^. The alula consists of 2–3 feathers at the leading edge of most birds’ wings^15,37^. On average, the alula size varies between one-fifth to one-tenth of the total span of a bird’s wing^15,37,38^. Birds with high wing loading or that frequently maneuver at high angles of attack are the birds with the most pronounced alula according to the weight, span, and alula measurements reported in reference^13^. Savile et al. also studied the evolution of the avian wing with a focus on the aerodynamic performance of different birds. They observed that birds mainly used their alulas during low-speed flight control for landing, takeoff, and perching maneuvers. The connection between the alular digit and the carpometacarpus allows complex motion of the alula, which facilitates the performance of such maneuvers. The alula moves both in-plane and out of the plane of the wing, meaning it can abduct, adduct, pronate, and supinate^15,37^. The alula can be found in insects and birds. We focus on birds’ alula in this section and insects’ alula in a later section.

Aerodynamically, the alula is a post-stall lift enhancement device studied in the literature through numerical simulations, wind tunnel testing, and flight observation of birds. Savile et al^13^. proposed that the mechanism by which the alula works is such that it acts as a leading-edge slotted flap that deploys passively by large birds or small active birds. The slotting accelerates the flow between the wing and the flap, which mixes and energizes the flow past the flap, thus delaying separation and increasing maneuverability. Multiple studies hypothesized that the deployment of the alula is passive^37,39,40^. However, Carruthers et al.^41^ observed the alula deployment through video recording and divided it into two stages: a) passive peeling in response to changes in the flow conditions, and b) active protraction of the alula through a 45^∘^ sweep forward to the leading edge^41^. Austin et al.^39^ went further and proposed an additional mechanism for the alula. They proposed that the alula suppresses the vortex bursting over the upper surface by generating vortical structures that maintain the lift over the hand wing. Lee et al.^42^ studied the alula based on flight observations and wind tunnel experiments. Using digital particle image velocimetry, Lee supported the hypothesis in ref. ^39^ and showed that the alula increases lift at high angles of attack (AoA) by working as a vortex generator (Fig. 2b-i).

In more recent years, a transition towards using engineering wings with alula-inspired devices occurred in order to systematically study the design parameters of the alula and their effect on aerodynamic performance, as well as to implement them as flow control devices on sUAVs (Fig. 2b-ii)). Mandadzhiev et al. mounted an alula-inspired device on an S1223 airfoil (i.e., a 2D experiment)^43^. Their study shows that the alula-inspired device enhanced lift at post-stall angles of attack and that the lift enhancements are a function of the device’s deployment parameters. Ito^44,45^ expanded on Mandadzhiev’s study^43^ by mounting the alula-inspired device on a finite wing rather than an airfoil (i.e., a 3D experiment). The study showed that the lift enhancements for the 3D experiment (37%) were more pronounced compared to the 2D experiment (8%). The additional lift enhancement was attributed to the hypothesis that the alula-inspired device works not only as a slotted flap but also that it generates a tip vortex that acts as a boundary layer fence preventing the propagation of stall outboard^44^. Thus, Ito’s^44,45^ results show that the alula is a 3D post-stall lift enhancement device, supporting biological observation in refs. ^39^^,^^37,46^. Linehan et al.^47,48^ used PIV to study the vortical structures created by the alula-inspired device and their interaction with the wing vortices. The authors proposed that the alula works through leading-edge vortex roll-up and tilting. More specifically, the alula causes a spanwise flow which energizes the shear layer and directs the flow towards the wingtip vortex, enhancing post-stall lift production^47,48^. In further work, Linehan et al.^38^ studied the scale effect and location of the alula, showing that a lift-maximizing position also corresponds to the actual evolutionary location of alula in birds.

Linehan and Ito studied the alula experimentally in a steady flow at Reynold’s number 75,000 and 100,000–120,000, respectively. Linehan studied the effects of the different parameters by modeling the wing and alula as flat plates. Ito et al. added complexity to the geometry using an S1223 airfoil and a bird-inspired alula profile. However, both studies lack the addition of flapping or aeroelastic effects. A study by Bao^49^ added the flapping complexity by studying a sinusoidal flapping input numerically on an alula similar in geometry and flow conditions to Ito. The numerical solver used Unsteady Reynold’s-Averaged Navier-Stokes with a *κ*–*ω* turbulence model. Results support that the alula works through slat and vortex interaction effects. Further, it shows that the slot effect dominates at the start of the upstroke. However, the vortex effect dominates in the midpart of the upstroke, and both play a role in the downstroke. The authors also report the effect of different design parameters on the alula trailing edge vortex and the alula streamwise vortex, affecting the lift response^49^. Finally, Zekry et al.^50^ used a design of experiment approach with linear regression models to produce response surfaces for lift and drag forces as a function of the flight conditions and the alula parameters, which can be later used in control laws for flight control systems.

In summary, the alula is a very effective high-lift device as it can maintain flow attachment over the wing at high AoA and low-speed flight. It accelerates the flow between the wing and the alula lower surface, energizing the flow and delaying separation. The alula also generates a vortex at its tip that acts as a boundary layer fence to prevent the propagation of stall outboard of the wing. Thus, we classified the flow control mechanisms for the alula and alula-inspired devices as separation control and vortex tailoring (Table 1). Furthermore, in addition to flow control, alula-inspired devices can be used for flight control on sUAVs if deployed asymmetrically on the left and right wings, as they can produce lift and drag differentials resulting in rolling and yawing moments^51^. Despite all these advantages, alula literature is still limited and the structure is yet to be implemented on flight vehicles. Alula-inspired devices should also be tested in flight on vehicles across various ranges of speeds and scales. Future work should also focus on characterizing the alula’s dynamics and aeroelasticity during gliding and flapping flights, since no papers discuss these issues.

### Slotted wingtips

Another local and passive feather flow control system in birds is the wingtip slots. In some species, the five to six primary feathers at the tip of the wings emarginate, or narrow down, forming slots or gaps near the wingtip. During flight, such slots cause the wingtip feathers to separate or spread horizontally and vertically^13,18^. Liu et al. present a detailed review of the aerodynamic effects of these wingtip slots on biological and engineered wings^52^. However, we will summarize here some of the key results and the enabling physics of these flow control devices.

Wind tunnel experiments of birds’ wings with the slotted wingtips have detailed the aerodynamic benefits of slotted wingtips^18,53–56^. Many of these studies noted that the slotted wingtips reduce the lift-induced drag of the wing. The drag reduction caused by slotted wingtips can be attributed to the shape of the wingtips during flight under aerodynamic loading. In flight, due to aerodynamic loading, the tips of the slotted wingtips bend upwards and separate vertically, while the broad root of the slotted wingtips overlap, limiting root twist and bending. The bending and separation at the tip of the slotted wingtips form a non-planar slotted wingtip configuration^18,53,54^. The non-planar wing can generate less induced drag and similar lift compared to a planar wing, that is straight from tip to tip, at the same speed^18,53^. For example, Tucker et al. show that the unclipped wingtips, which form a non-planar configuration, have 70% to 90% of the drag of the clipped wingtips, which form a planar configuration^53^. Hence, slotted wingtips can improve aerodynamic efficiency, defined as the ratio of lift to drag, by generating a similar lift with less drag than a wing without slots.

Flow visualization was also used to understand the effect of the wingtip slots on the flow field and tip vortices around birds’ wings. KleinHeerenbrink et al. used particle image velocimetry to measure the airflow around the slotted wingtip of a jackdaw (*Corvus monedula*) in a wind tunnel during gliding and flapping flight. Their results confirm that the separated primary feathers produce individual wingtip vortices, spreading the overall wingtip vorticity in the horizontal and vertical planes, which was previously associated with improved efficiency^54^. March et al. also used flow visualization around the wingtips of a Great Horned Owl (*Bubo virginianus*). Their results show smooth streamlines near the slotted wingtips^55^. Thus, these flow visualization studies suggest that the slotted wingtips spread the vorticity behind the wings, decompose the upwash from the tip vortex, and reduce the induced drag (Fig. 2c-i,iii)^54,55^.

Researchers continued to study the physics of the slotted wingtips through the application and study of engineered wingtips/wingtip sails. Wingtips or wingtip sails are small devices attached to the tip of engineered wings, inspired by the slotted wingtips, to enhance the aerodynamic performance^57–59^. Some of the wingtip parameters explored in the literature include the number of wingtips, the dihedral, twist, incidence angles of individual wingtips, and the gap between the wingtips. Results show that using multiple wingtips with various dihedral angles reduces the overall tip vortex by splitting it into smaller vortices and reducing the effective downwash at the wing (Fig. 2c-ii,iv). These effects often resulted in increased aerodynamic efficiency and delayed stall without incurring considerable lift penalties^52,57–67^. Negative incidence angles have increased the aerodynamic efficiency by re-orienting the lift vector forward and counteracting the drag^62,63,68^. Finally, the increase in the gap distance between the wingtips was shown to weaken the tip-vortex suppression^52,65^, suggesting the importance of tuning the gap size to enable vorticity spreading and wingtip vortex suppression.

Thus, in both biological and engineering studies, the flow control mechanism for the slotted wing tips is vortex tailoring because they mainly spread the wingtip vortices (Table 1). Such a flow control mechanism was also implemented for flight control. The induced drag effect of the wingtips was shown to provide yaw stability when implemented asymmetrically on the left and right wings^56^. Some researchers have also applied wingtip slots/sails to aircraft during flight^52,59^. In general, flight testing results confirmed the wind tunnel results, indicating that slotted wingtips or wingtip sails can be used to improve aerodynamic efficiency by reducing drag compared to a planar or a conventional wingtip.

Despite the numerous studies on the aerodynamic effects of wingtip slots, an optimal configuration across all vehicles or systems does not exist since the aerodynamic benefits of using engineered wingtips depend on the in-flight conditions such as the Reynolds number and angle of attack. There is also a need for an adaptive slotted wingtip system^62,67,68^. An adaptive system is needed to adjust the wingtip dihedral angle, incidence angle, and gap space as a function of the flight conditions to yield the desired aerodynamic effects. Additionally, most of the research on engineered wingtips was conducted in wind tunnels and through numerical simulations, rather than flight tests on actual flight vehicles. Hence, more studies on the applications of engineered wingtips are required to assess the in-flight aerodynamic performance for future research^59,69^.

### Leading edge serrations

Leading edge serrations (LES), defined as comb-like hooks structures that exist on the outer vanes of the feathers at the leading edge of the wing, and trailing edge fringes, defined as unconnected barbs at the ends in feathers, have been studied extensively for noise reduction and silent flight, especially in owls^14,70–76^. However, given the focus of this article on flow control, rather than noise suppression, we will highlight the few studies related to the role of leading edge serrations on aerodynamic tailoring.

Alongside their noise-canceling capabilities, LES can be considered flow control devices for their role in improving aerodynamic efficiency and tailoring laminar to turbulent transition^70,72,77^. In 1971, Kroeger et al.^74^ used flow visualization on two prepared owl wings and found that LES act as co-rotating vortex generators, which stabilize the flow over the upper surface of the owl’s wing and prevent laminar separation. As a result, these vortices are able to drive higher momentum flow towards the wall surface, which can also delay stall, similar to vortex generators. These findings inspired engineering studies using similar serrations designs on traditional airfoils and rotor blades to test their functionality as a passive flow control device. In the post-stall regime, multiple studies show that the LES can effectively improve the aerodynamic performance of the stalled airfoil by increasing lift, reducing drag, and reducing the fluctuation of aerodynamic forces^78–80^. Wenzen et al. and Rao and Hiu^81,82^ studied the Reynolds number dependency of LES and found that they are effective as post-stall flow control devices for *Re* ≥ 40, 000. Muthuramalingam et al.^83^ studied the LES effect on 3D backward swept wings and found that the serrations have a flow-turning effect that counteracts the outboard cross-span flow that typically appears for swept-back wings. This effect can attenuate cross-flow instabilities and delay laminar to turbulent transition.

Leading-edge serrations implement various flow control mechanisms, including transition and separation control, as well as vortex tailoring (Table 1). However, despite their potential as flow control devices, there is still no full understanding of the exact physical mechanism by which LES mitigate stall and tailor transition, preventing implementation on existing UAVs. Moreover, most studies on LES have been conducted in low-turbulence acoustic wind tunnels, which differs from real scenarios in which turbulence may have a notable impact. Finally, the interaction between LES and other traditional or bio-inspired flow control techniques has not been fully studied, so there is a lack of understanding of how LES would alter the flight envelope or stability parameters of an actual flying vehicle.

## Insect-scale flow control

Insect wings generate aerodynamic forces to accelerate or to stay aloft in the air. Unlike flying vertebrates, like birds and bats, which use limbs and muscles to control their wing shape, the muscles of insects do not extend beyond the body. Hence, the shape of the insect wing is controlled passively as its flexible wing constantly deforms in response to in-flight forces^84–86^.

### Wing corrugations

One of the main features of insect wings is corrugation. Wing corrugations provide aerodynamic benefits by controlling airflow, and structural benefits by withstanding aerodynamic loads during flight^87,88^. This review mainly focuses on the aerodynamic role of wing corrugations.

**Fig. 3:**
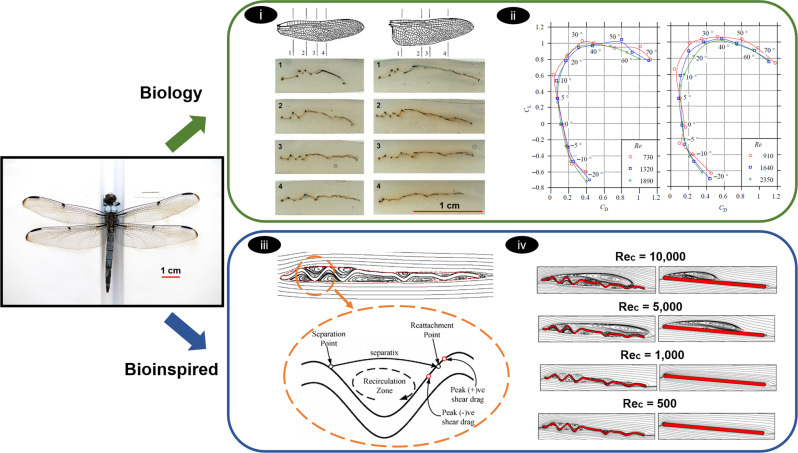
Summary of biological and bioinspired studies on the role of wing corrugations in insects. There have been several (top) biological and (bottom) bioinspired studies on insect wings especially focusing on the role of corrugations. (i) Wing corrugations in the hind (left) and fore (right) wings of dragonflies vary along the chord and span [adapted with permission from ref. ^93^]. (ii) Drag polar diagrams from wind tunnel force measurements of fore (left) and hind (right) wings of a dragonfly at different Reynolds numbers showing high L/D ratio across the range of Reynolds number tested [adapted with permission from ref. ^98^]. The effect of insect wing corrugations has also been studied on engineered wing sections (iii and iv). (iii) Corrugations trap vortices in the peaks and valleys of the wing profile. Trapped vortices energize the boundary layer and mitigate flow separation. (iv) Compared to a flat plate profile (right), the corrugated profile (left) produces 32% higher L/D [adapted with permission from ref. ^103^].

There have been several morphological studies focused on characterizing the corrugations of insect wings using 3D reconstruction of cross-sectional 2D imaging (photogrammetry)^89–95^ and 3D scanning (micro-CT) techniques^96^ (Fig. 3-i). Morphological studies on the wings of dragonflies, damselflies, hoverflies, butterflies, locusts, fruit flies, blowflies, and beetles show that the wings of the insects are corrugated and their wing cross sections at different spanwise locations display varying corrugation patterns^89–97^. Following the morphological studies, there have been a few wind tunnel experiments conducted on the actual insects to study their wing’s aerodynamics. Experiments on dragonflies show an increase in maximum lift coefficient and maximum lift-to-drag ratio compared to a flat plate (Fig. 3-ii)^93,98^. However, studies on actual insect wings are limited, given their scale and measurement limitations. Thus, most studies on the aerodynamic effects of corrugations are performed either numerically or experimentally on engineered wing sections with corrugation profiles measured from morphological studies.

While the aerodynamic properties of several insect species (e.g., dragonflies, damselflies, hoverflies, butterflies, locusts, fruit flies, blowflies, and beetles) have been investigated, most studies have focused on the corrugated wings of dragonflies since they exhibit higher aerodynamic efficiency (lift to drag ratio) compared to those of other insects^89–92,94–97,99–105^. Results from the aerodynamic force measurements show that the corrugated wings of dragonflies generally have higher aerodynamic efficiency (lift-to-drag ratio) compared to a flat plate and streamlined airfoils^100–102^. For instance, a corrugated profile based on dragonflies wing generates 32% and 5% higher lift to drag ratio compared to a flat plate and the streamlined airfoil, respectively at *α* = 5^∘^ and *Re* = 10,000^103^. Studies show that the aerodynamic enhancements are due to the increase in lift^92,100,102^ and drag reduction^101^.

Flow visualization experiments, such as PIV, and numerical simulations have also been used to analyze the flow fields around corrugated wings. The results from the experiments and simulations on the corrugated wing sections of dragonflies, hoverflies, butterflies, and locusts show that the vortices generated by corrugated wings play an important role in delaying flow separation^89–91,94,101–106^. Results show vortices trapped in between the peaks and valleys of the corrugated wings (Fig. 3-iii, iv). These vortices induce high-velocity flow, energizing the boundary layer, and mitigating flow separation^94,101,102,104^. In addition, the vortices between the peaks and valleys alter the effective wing profile making it more airfoil-like. As the corrugations are filled in with recirculating vortices, the wing maintains a laminar boundary layer, and the shear drag of the system is reduced. Shear drag is a major contributor to the total drag at a lower Reynolds number, as viscous effects are more dominant. At Reynolds number of 10,000, the shear drag produced by the corrugated airfoil constitutes 24% of the total drag, in contrast to 65% for a smooth airfoil or a flat plate^103^. In summary, the thin and lightweight corrugated wings of insects act like a profiled cross-section for aerodynamic benefits with the advantages of low mass, reduced overall drag, and delayed separation.

In contrast to studies that support the aerodynamic role of the corrugates, a few studies disagree with the aerodynamic benefits of corrugations^89,91,94,105,107^. Experimental and numerical studies found that structural corrugation does not significantly impact aerodynamic performance^89,94,105,107^. The corrugated and flat plates produced similar aerodynamic forces, and some even show that the corrugations increased drag at the peak gliding ratios. Flow simulations have demonstrated that corrugated wings produce high-pressure drag compared to flat plates due to the increase in the thickness of the viscous region^91,105,107^.

Studies thus far have focused on the static aerodynamic effects of corrugations. However, insects mainly use flapping flight for locomotion. Hence, there is a need for experiments and simulations focused on the role of corrugations during flapping to implement such profiles on insect-scale robots. Moreover, corrugated insect wings undergo deformation due to their flexibility during flapping flight, and limited studies have investigated the combined effects of the aerodynamic, flow control effects, flexibility, and deformation of flapping corrugated wings^108–112^. Therefore, studies incorporating wing kinematics, flexibility, and deformation are needed to understand the aerodynamic effects of corrugated wings better.

### Insect alula

Another local flow control device that has been investigated for insects is the alula. The alula in insects is a hinged flap found at the base of the wings of some insects. The alula is actuated via a component at the wing hinge. During flight, the alula can either be flipped or flat^113^. In their study, Walker et al. studied the wing beat cycle of hoverflies with the alula in the flipped or flat states. The alula state was associated with different wing kinematics. The study concluded that the alula state might indicate the flight mode, and that when the alula is flipped, the wings produce less aerodynamic forces^113^. In a more recent study, numerical simulations were used to investigate the role of the alula on a simplified rectangular wing. Their results show that the wings with the alula require less aerodynamic power than those without the alula. The authors also show that the phasing between the wing and the alula matters. The wing with alula flapping in phase produces the largest lift, but the efficiency is the lowest. The alula also provides a stabilizing effect on the leading edge vortex for the wings when the alula flaps 45^∘^ ahead or in phase^114^.

Table 1 highlights some studies on insect flow control devices. The flow control mechanism for the wing corrugations combines all three mechanisms identified in this article, while the alula’s mechanism can be classified as mainly vortex tailoring. Compared to avian and avian-inspired flow control devices, insects’ passive and local flow control devices are less studied, except for the wing corrugations. There have been few implementations of insect-inspired flow control devices on engineered vehicles. Current flying insect-scale robots have mostly flat plate wings, with most focusing on designing actuation mechanisms at the root of the wing. Therefore, future research on the application of insect wings’ corrugations, flexibility, and deformations on insect-scale robots can be used to understand insect flight better and enhance current robotic wings with the aerodynamic and structural benefits highlighted by the previous research^115,116^.

## Aquatic bio-inspired flow control

**Fig. 4:**
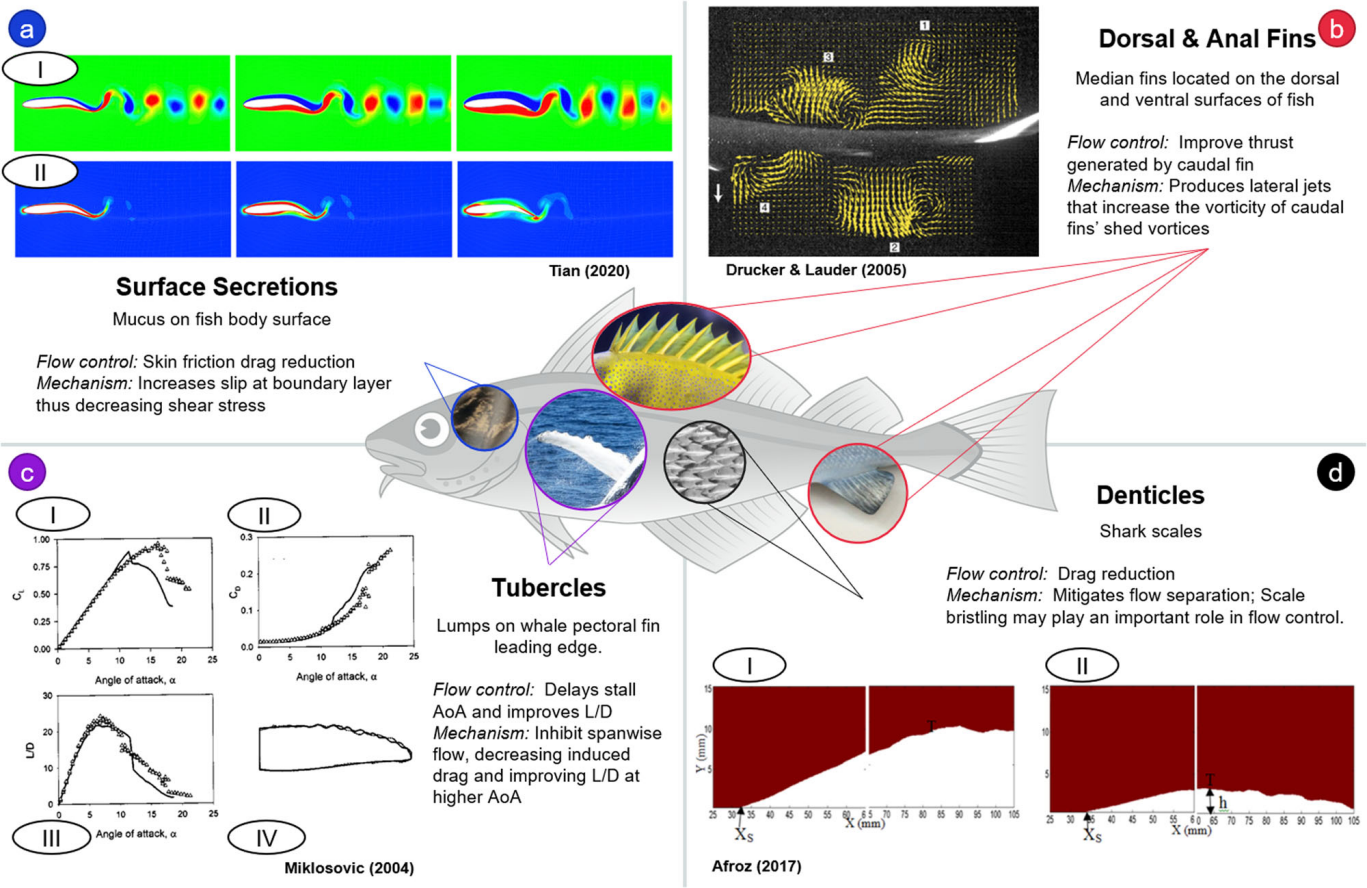
A summary of major flow control strategies found in swimming organisms. A model swimmer is presented in the center as a generic fish^163^ [source: Pixabay], with specific examples of aquatic flow control devices indicated by the inserted graphics^144,164^. **a** Surface Secretions^157^ [source: Pixabay]- A 2D simulation of a fish represented by a deforming flapping foil (top view) in fluids of varying viscosity. From left to right, the columns show data from a shear-thinning fluid, a Newtonian fluid, and a shear-thickening fluid. The shear-thinning fluid represents a simplified consistency of fish slime. (I) The wake of the foil in the shear-thinning fluid indicates thrust rather than drag, which is seen in the other two fluids. The colors indicate vorticity, where blue indicates a non-dimensional value of −5 and red indicates 5. (II) Plotted are contours for the second invariant of rate of the strain tensor, where blue and red indicate non-dimensional values of to 400, respectively. The boundary layer is seen to be thinner for the shear-thinning fluid, suggesting reduced drag^156^. **b** Dorsal^125^ and Anal^126^ Fins, [source: Pixabay]- PIV visualizations of lateral momentum transfer jets induced by dorsal and anal fins of a rainbow trout (top view). Shown is the wake of the dorsal fin (left edge) as it approaches the caudal fin (right edge). This wake, which is the incident flow seen by the caudal fin, contains alternating vortices which are markedly different from the freestream. Jets are also visible in the wake, directed laterally for stability^124^. **c** Tubercles^133^ [source: Pixabay]- Comparison of lift coefficient, *C*_*L*_, drag coefficient, *C*_*D*_, and lift-to-drag ratio, *L*/*D*, results from steady wind tunnel experiments for a whale flipper model with tubercles (open circles) and one without tubercles (closed circles). Stall is delayed by ~5° in the model with tubercles. At higher angles of attack, *AoA*, lift is higher, drag is lower, and thus *L*/*D* is higher for the fin with tubercles^132^. **d** Denticles^146^ [reproduced from ref 146, 2019, Biomimetics]^146^ Time resolved-DPIV results for flow over a plate without shark skin (I) and one with shark skin (II). The shark skin covered plate causes transition to occur sooner, reattachment to occur more rapidly, and induces a smaller laminar separation bubble^141^.

Flow control from aquatic creatures can be attributed to devices such as 1) fins, 2) secretions, and 3) surface textures. This is summarized in Fig. 4, which details some of the most studied aquatic flow control devices, including dorsal/anal fins on fishes, mucus secretions on fish bodies, denticles on shark skin, and tubercles on whale flippers. A few other, less studied flow control devices are mentioned briefly at the end of this section. Fish and Lauder performed an in-depth review of flow control in swimming animals in ref. ^117^, and much work has been done since then. This review provides a broad overview of aquatic flow control from a bioinspired perspective while building on the previous review with more recent studies.

### Fins

Fish fins have a variety of uses, such as sensing, locomotion, and flow control^118^, but when it comes to flow control, the median fins play the most significant role in many fish^119^. The median fins in fish include the dorsal and anal fins, which are centered along the mid-lines of the dorsal and ventral surfaces of many fish^120,121^. The wakes of these fins determine the nature of the incident incoming flow seen by the caudal fin, a median fin primarily used for thrust generation. Depending on the fish species and the swimming speed, the dorsal fin may undulate and generate its own vortical wake with an average velocity higher than that of the free-stream^122^. This wake then becomes the incoming flow seen by the caudal fin and is hypothesized to increase the momentum shed in the caudal wake^123^ (Fig. 4b)^124–126^. PIV visualizations of the dorsal fin wake also show lateral jets that are suggested to be used for stability^124,127^. While studies have mostly been performed on dorsal fins, anal fins have been shown to behave nearly symmetrically to the dorsal fins in some species and thus have similar impacts on the caudal fin incident flow. These fins often work together such that the rolling torques generated by one stabilize those generated by the other^127^. A notable robotic implementation of dorsal and anal fins for thrust improvement is seen in ref. ^128^, where model dorsal and anal fins are mounted to a robotic fish. Linear acceleration of the mechanical model was improved by up to 32.5% when the fins were added. Another example is a snake-like swimming robot whose swimming speed is observed to change depending on parameters including fin spacing and the amplitudes and angular velocities of the oscillatory swimming waveform^129^. Several other robotic dorsal/anal fin applications exist but are focused mainly on their role in stability^120,130^, rather than flow control.

### Surface structures

In whale fins, “tubercles” are the structures of interest for flow control. Tubercles are lumps located on the leading edge of their pectoral fins^131^. These lumps have been shown to help keep the flow attached at higher angles of attack, thus delaying stall and improving the lift to drag ratio at these angles of attack (Fig. 4c)^132^^133^. Tubercles improve the maneuverability of whale fins, which perform less complex 3D kinematics compared to ray-finned fishes, by giving them a greater range of mobility^119^. It is important to note that whales swim within a higher Reynolds number regime (around 10^6^) than many fish do (as low as 10^3^)^132^. Experimental^134^ and numerical^135^ studies on fins with tubercles in low Reynolds number regimes of approximately *Re* < 300,000 found that tubercles degraded rather than improved the hydrodynamic performance of the foils. Tubercles have been investigated on several airfoils showing various aerodynamic and hydrodynamic benefits. These studies are summarized in refs. ^136–139^. Most of these studies have been conducted in steady flow conditions. Thus, there is a dearth of studies performed on the influence of tubercles when the flow is unsteady. Such studies would greatly benefit the bio-inspired swimming community, as unsteady flow is a critical component of biologically inspired swimming.

Another widely studied surface feature for aquatic flow control, primarily known to reduce drag, is shark scales, also known as “denticles”^140^. Each denticle has a ridge known as a “riblet” that is aligned with the body axis of the shark, creating a rough texture on the shark skin^141^. Riblets have been observed to decrease turbulent skin friction drag in steady flow independently of whether they are on denticles or a smooth surface^142^. Denticles are also known to bristle, a movement that can be described as varying their pitch angles relative to the shark surface. The bristling is hypothesized to reduce pressure drag by inhibiting backflow in the boundary layer^143^. For an accelerating foil, a denticle-like texture was found to have delayed boundary layer separation compared to a foil with no texture^144,145^ (Fig. 4d)^141,146^. Wen et al.^147^ presented 3D-printed shark denticles that can be easily modified and applied to surfaces. Others have studied aspects of biomimetic denticles, such as swimming performance and boundary layer separation on an accelerating body^148,149^. Still, there is a lack of research on denticles directly applied to swimming robots.

### Mucus

The final flow control device studied in aquatic organisms is secretion. Many fish secrete mucus that covers their bodies for a variety of uses, one of which is flow control^150–152^. Studies show that the non-Newtonian mucus layer acts as a buffer between the solid fish surface and the surrounding water, effectively creating a slip condition between the fish and the water^153,154^. This is hypothesized to reduce shear at the boundary layer, thus decreasing skin-friction drag^155^ (Fig. 4a)^156,157^. In one specific case study, the mucus layer in puffer fish is observed to decompose as velocity increases. However, drag is still reduced due to the newly uncovered tips of their spines that texture the surface of the fish. Thus, the mechanism changes from reducing skin-friction drag via a slip layer to reducing pressure drag via the textured surface^153^. The mucus layer as an engineering flow control strategy is problematic due to the gradual degradation of the layer in the presence of an external flow^156^. To combat this issue, Lee et al. presented a slippery lubricant-infused surface, “LIS,” which was shown to be robust when exposed to steady shear from the surrounding fluid while reducing friction drag by 18%^150^. Again, it would be interesting to see how this fluid withstands shear in accelerating external flows similar to the ones experienced by fish.

Other flow control strategies that are not discussed in detail in the present work include several studies on dolphins, which are known to be highly efficient swimmers. Wainwright et al.^158^ demonstrated that ridges on dolphin skin may improve sprinting performance, but the data is not conclusive. Another recent study on the color of dolphin skin strongly suggests that darker colors on the dorsal surfaces of swimmers can reduce skin friction in turbulent conditions due to thermal effects^159^.

Table 1 summarizes some studies on aquatic flow control devices. The flow control mechanisms of such devices vary from separation control (e.g., tubercules), to vortex tailoring (e.g., fins) and transition control (e.g., denticles), and some of these devices implement more than one flow control mechanism. Most of these aquatic flow control devices have not been implemented on unmanned aquatic vehicles or fish-inspired robots, creating research opportunities towards improving the performance of the bioinspired systems.

## Outlook

This article highlighted several passive and local biological and bioinspired flow control devices for birds, insects, and aquatic animals. The number of species and animals that inspire these flow control devices shows the efficiency of these devices in favorably altering the flow across scales and locomotion regimes. Even though flow control devices in nature have different morphologies and manifestations, they all modulate the flow through three common mechanisms: separation control, transition control, and vortex tailoring. The studies highlighted in this article also showcase the diverse research methods involving these devices, ranging from experimental studies using wind tunnels and free-flight tests to numerical simulations and analytical models.

The article also identified research gaps for each flow control device. However, some gaps are related to most of the flow control devices discussed in this article. For example, few studies have considered the fluid-structure interaction between these devices and the unsteady aerodynamic flow surrounding them. Some studies have also limited this interaction by assuming the device is rigid rather than flexible or deformable under aerodynamic or hydrodynamic forces. Furthermore, the lack of consistency across studies in terms of flow regimes, Reynolds numbers, and the adaptation of the bioinspired flow control devices make it difficult to perform quantitative comparisons between the studies. Another gap involves simplifying or eliminating the multi-body dynamics of these locomotion strategies. For example, most studies on the coverts feathers, the insect wing corrugations, and the shark-inspired denticles have ignored the wing dynamics (i.e., flapping) or the body and fin oscillations. These gaps create future research opportunities and allow for interdisciplinary collaboration among biologists, fluid mechanicians, dynamists, and controls engineers. Such collaborations can improve our understanding of the physics of biological flow control devices and allow further implementation of these devices on engineered vehicles.

Despite the research gaps, the recent increase in studies focused on bioinspired flow control devices indicates that such devices offer unique opportunities for enhancing the aerodynamic and hydrodynamic performance of engineered systems beyond traditional flow control devices. There have been notable successes in implementing bioinspired flow control devices, especially on flight vehicles showcasing lift enhancements, drag reductions, and improved aerodynamic efficiency across various speeds and vehicle configurations. Thus, bioinspired flow control is a feasible approach for improved performance in engineered vehicles. With proper cost-benefit, sizing, and performance analyses, such devices can be fully and routinely integrated within aerial and aquatic vehicles.
