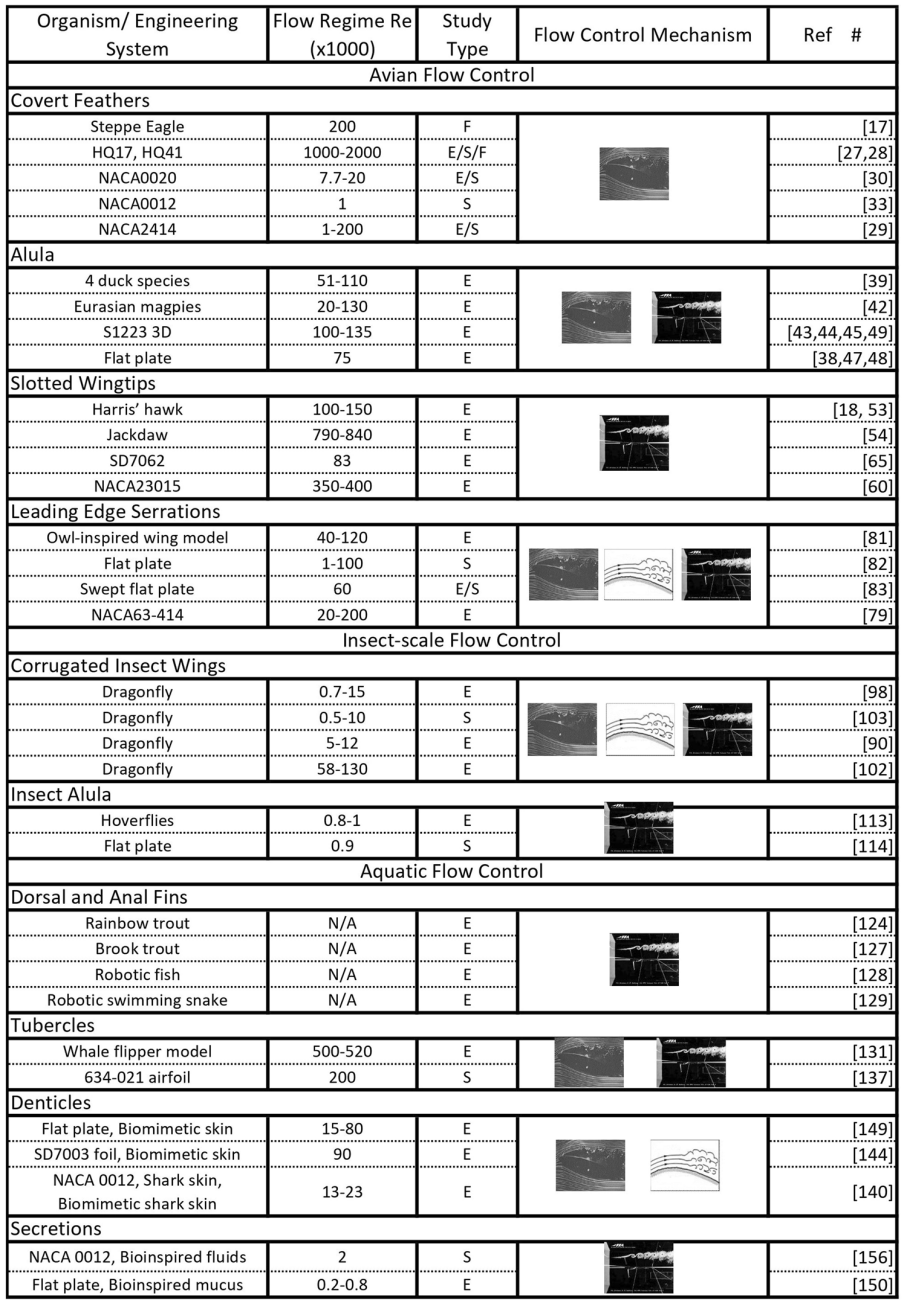# Author Correction: Aerial and aquatic biological and bioinspired flow control strategies

**DOI:** 10.1038/s44172-023-00094-z

**Published:** 2023-07-11

**Authors:** Ahmed K. Othman, Diaa A. Zekry, Valeria Saro-Cortes, Kyung Jun “Paul” Lee, Aimy A. Wissa

**Affiliations:** Department of Mechanical and Aerospace Engineering, Princeton University, Princeton, NJ 08544 USA

**Keywords:** Mechanical engineering, Physiology

Correction to: *Communications Engineering* 10.1038/s44172-023-00077-0, published online 26 May 2023.

Author correction:

In the original version of this review, the citation information in the legend to Figure 1 and in Table 1 was incorrect. In addition the title for Table 1 had a typographical error. The revised versions are below and the items have now been corrected in the PDF and HTML versions of the review.

Fig. 1 Flow control mechanisms and classifications and their implementations as flow control devices.

The superscripts indicate the flow control mechanism implemented by each flow control device. The figure classifies flow control mechanisms into 1- separation control [Reproduced from ref. ^160^, The National Aeronautics and Space Administration (NASA)]^160^, 2- transition control [Reproduced from ref. ^161^, The National Aeronautics and Space Administration (NASA)]^161^, 3- vortex tailoring [Reproduced from ref. ^162^, The National Aeronautics and Space Administration (NASA)]^162^.

Table 1: